# PD82 Patient Or Public? A Case Comparison Of Stakeholder Contributions In Health Technology Assessment

**DOI:** 10.1017/S0266462325102092

**Published:** 2025-12-29

**Authors:** Keunjoo Yoo, Sung-Hee Hwang, Sangjin Shin, Sumin Eom, Jieun Choi

## Abstract

**Introduction:**

In South Korea, health technology assessment (HTA) evaluates the safety, efficacy, and cost effectiveness of technologies reimbursed by national health insurance. The National Evidence-Based Healthcare Collaborating Agency (NECA) engages the public and patients in HTA to enhance transparency and relevance. This study examined intra-articular corticosteroid injections (IACS) for knee osteoarthritis and radiofrequency ablation (RFA) for thyroid nodules to illustrate the role of diverse stakeholder input in decision-making.

**Methods:**

Public Participation for IACS: IACS is a familiar treatment for knee osteoarthritis in Korea and has been used extensively for decades. To capture public perceptions, a structured survey was conducted to identify concerns about treatment frequency, safety, and efficacy. Findings were synthesized with systematic reviews to create public-friendly materials such as infographics, videos, and social media content.

Patient Participation for RFA: RFA, a non-reimbursed procedure for thyroid nodules, required direct input from patients to assess its value. Semi-structured interviews with RFA recipients explored their decision-making priorities, such as symptom relief, cosmetic concerns, and post-procedure recovery. These insights informed the evaluation framework for RFA in HTA.

**Results:**

Public surveys on IACS emphasized the need for clearer guidelines about dosing and safety, particularly for repeat injections. Systematic reviews showed limited evidence on long-term adverse effects, prompting further research. Educational materials enhanced public awareness of risks and benefits, supporting informed choices.

Interviews with patients who had received RFA revealed unique challenges and shared concerns specific to thyroid conditions, such as post-procedure discomfort and symptom management priorities. These insights, including preferences for nodule size reduction and recurrence rates, aligned with expert-defined outcome priorities, validating patient-reported outcomes in HTA. These findings led to a conditional recommendation balancing clinical value and misuse prevention.

**Conclusions:**

Integrating public and patient voices into HTA strengthens evidence generation and fosters trust in health policy decisions. By addressing diverse stakeholder concerns, NECA aims to expand engagement mechanisms throughout the HTA life cycle. This dual-participation model demonstrated a scalable framework for global HTA practices, promoting inclusivity and transparency in healthcare decision-making.

